# Evaluation of caries risk reduction following preventive programs in orthodontic patients, using Cariogram computer model: A quasi-experimental trial

**DOI:** 10.1590/2177-6709.26.5.e2120218.oar

**Published:** 2021-10-25

**Authors:** Maryam DOOST-HOSEINI, Massoud SEIFI, Mina PAKKHESAL, Abolfazl SABOURY, Parisa AMDJADI, Aliakbar NAGHAVIALHOSSEINI

**Affiliations:** 1 Shahid Beheshti University of Medical Sciences, School of Dentistry (Tehran, Iran).; 2 Shahid Beheshti University of Medical Sciences, School of Dentistry, Department of Orthodontics (Tehran, Iran).; 3 Golestan University of Medical Sciences, School of Dentistry, Department of Community Oral Health, Dental Research Center (Gorgan, Iran).; 4 Shahid Beheshti University of Medical Sciences, School of Dentistry, Department of Prosthodontics (Tehran, Iran).; 5 Shahid Beheshti University of Medical Sciences, School of Dentistry, Department of Dental Biomaterials (Tehran, Iran).; 6 Golestan University of Medical Sciences, School of Dentistry, Department of Orthodontics, Dental Research Center (Gorgan, Iran).

**Keywords:** Caries risk assessment, Preventive, Orthodontic, Cariogram

## Abstract

**Objective:**

This study evaluated the effectiveness of preventive strategies on caries risk reduction in patients undergoing orthodontic treatment, using the Cariogram program.

**Methods:**

In this quasi-experimental study, samples were selected using a convenience quota sampling technique, in a public dental school. At first, caries risk profile was determined for each subject using the Cariogram before brackets bonding. The sample size consisted of 36 patients. The intervention group (n = 18) received preventive programs, and the control group (n = 18) was trained based on the routine oral health education by means of pamphlets. Then, Cariogram parameters were calculated for patients in both groups after six months.

**Results:**

The age range of participants was from 12 to 29 years. The mean percentage of the *“Actual chance of avoiding new cavities”* section in the intervention group increased from 45.72 ± 21.64 to 62.50 ± 17.64. However, the mean percentage of other parameters - such as *“Diet”*, *“Bacteria”* and *“Susceptibility” -* decreased after six months (*p*< 0.001). Besides, the differences in the mean percentage between intervention and control group at the end of the study period (T_1_) related to the Cariogram parameters were statistically significant (*p*< 0.001). Accordingly, the mean percentage of *‘Actual chance of avoiding new cavities’’* parameter in the intervention group (62.50) was statistically higher than in the control group (42.44) (*p*< 0.001).

**Conclusion:**

Implementing different preventive approaches is able to reduce the caries risk in patients undergoing fixed orthodontic treatment, which can be clearly demonstrated using Cariogram program.

## RESUMO

**Objetivo:**

O presente estudo usou o *software* Cariogram para avaliar a eficácia de estratégias preventivas para redução do risco de cáries em pacientes sob tratamento ortodôntico.

**Métodos:**

Nesse estudo quase-experimental, as amostras foram selecionadas por meio de uma técnica de amostragem por cota de conveniência, em uma faculdade pública de Odontologia. Inicialmente, o perfil de risco de cárie foi determinado para cada indivíduo usando o Cariogram antes da colagem dos braquetes. A amostra consistiu de 36 pacientes: o grupo experimental (n = 18) recebeu programas preventivos, e o grupo controle (n = 18) recebeu orientações sobre a saúde bucal por meio de folhetos. Após seis meses, os parâmetros obtidos por meio do Cariogram foram calculados novamente para os pacientes de ambos os grupos.

**Resultados:**

A faixa etária dos participantes foi de 12 a 29 anos. A porcentagem média da seção *“Probabilidade real de prevenir novas cáries”* no grupo experimental aumentou de 45,72 ± 21,64 para 62,50 ± 17,64. Por outro lado, a porcentagem média de outros parâmetros - como *“Dieta”*, *“Bactérias”* e *“Suscetibilidade” -* diminuiu após seis meses (*p*< 0,001). Além disso, as diferenças nas porcentagens médias entre o grupo experimental e o grupo controle ao fim do estudo (T_1_), relacionadas aos parâmetros do Cariogram, foram estatisticamente significativas (*p*< 0,001). Assim, a porcentagem média do parâmetro *“Probabilidade real de prevenir novas cáries”* no grupo experimental (62,50) foi estatisticamente maior do que no grupo controle (42,44) (*p*< 0,001).

**Conclusão:**

A implementação de diferentes abordagens preventivas pode reduzir o risco de cárie em pacientes sob tratamento ortodôntico com aparelhos fixos, o que pode ser observado claramente por meio do *software* Cariogram.

## INTRODUCTION

Oral cavity is the habitat of various bacterial species, mycoplasma, protozoa, and yeasts, and any external interference can disturb the balance of microbiota in this environment.^1^ The traditional concept of caries as a multifactorial transmittable and infectious disease has been questioned. The current etiological concept of dental caries has emphasized the important role of sugars in caries. The current definition points toward an ecological disease caused by the commensal microbiota that, under ecological imbalances, mainly due to high and or frequent sugars consumption, creates a state of dysbiosis in the dental biofilm. It is currently accepted that caries is a sugars and biofilm-dependent disease. Acid-producing bacteria and other factors facilitate the development of dental caries. Also, salivary flow, fluoride exposure, plaque accumulation, tooth morphology and structure would create more favorable or adverse conditions for the causal relation between sugars and the dental biofilm to induce carious lesions.^2^

The development of dental caries is determined by the balance of protective and risk factors. If the dentist can recognize the relationship between these factors and the development of the disease or its relapse, the risk of caries will be reduced.^3^ ^,^ ^4^ Environmental, behavioral, and biological factors can be identified as risk factors associated with the incidence of the disease.^5^ Fixed orthodontic appliances such as brackets are examples of environmental factors. They are associated with increased plaque accumulation around the brackets and thus increase the burden of *Streptococcus mutans* and *lactobacillus* contamination in saliva and biofilm.^6^ The introduction of fixed appliances into the oral cavity not only intensify the amount of biofilm formation, but also increases the level of acidogenic bacteria inside the biofilm, resulting in a higher cariogenic challenge around orthodontic brackets and bands. If patients cannot maintain good oral hygiene during orthodontic treatment, the acid produced by dental biofilms will eventually lead to enamel demineralization and white spot lesions.^7^ ^,^ ^8^

Caries Risk Assessment (CRA) is an important phase in dental treatment based on the strategy of minimally invasive therapy, in which therapeutic and prophylactic measures are planned, based on the results of CRA.^9^ There is a number of available questionnaires and tests that first identify the level of risk exclusively for each patient, and allocate that individual into one of these three categories: low risk, moderate risk, or high risk. Cariogram model evaluates the data based on its algorithm and presents the results as a circular color chart representing five different groups of indicators, including: “Actual chance to avoid new cavities”, Diet, Bacteria, Susceptibility, and Circumstances.^10^ ^,^ ^11^

Next, appropriate preventive interventions may be done for each orthodontic patient. They can be motivated through regular stimulations that can encourage healthy behaviors in them. Reinforcement is one of the most important bases of health education, which helps patients to adopt healthy behavior and lifestyles. Text message reminder is able to improve the oral hygiene of patients undergoing orthodontic treatment.^12^

Therefore, we attempted to assess the effect of preventive strategies on reduction of caries risk in the intervention group. The present study was an experimental clinical research that analyzed all parameters of the Cariogram program, to evaluate the risk of caries in orthodontic patients treated with fixed appliances.

## MATERIAL AND METHODS

The present study was approved by the regional Research Ethics Committee (IR.SBMU.RIDS.REC.1395.250) and performed in complete accordance with the Declaration of Helsinki. Written informed consent was taken from the patients before the start of the research. Moreover, the data was handled anonymously and with confidentiality in all stages of the study. The researcher handled the data pseudonymized in the present study to protect the privacy of study participants while collecting, analyzing, and reporting data. The method of pseudonymization comprised separating identifying personal data from the questionnaire and preserving it with participants’ dental charts. In other words, two-time points Cariogram questionnaires were linked using a unique identification code allocated to each participant.

The sample size was calculated based on the data obtained from a previous study^13^, keeping a significance level of 0.05, standard deviations within groups of 30 units, a least detectable difference of 20 units between groups on the Cariogram, and power for that detection of 80%. Therefore, the sample size for each group was determined to be 18. Since there were two groups (intervention and control), the final sample size was determined to be 36.

Sampling was done using a quota sampling technique, in which samples were assigned from each caries risk profile (low, moderate, and high) until the sample met the minimum requirement in each study group. 

Inclusion criteria comprised orthodontic patients over 12 years old, with the ability to speak and understand the native language, and who needed fixed orthodontic treatment in both arches for at least six months. Exclusion criteria were: moderate or severe periodontal disease, cleft lip and palate or syndromic disorders, systemic diseases, and smoking or medications that could change the oral normal flora or the amount of saliva flow. Overall, the present study consisted of four phases:

I: Caries risk profile was determined for each subject using the Cariogram program. The caries risk profile for each participant was obtained on the basis of the magnitude of the sector *“Chance to avoid new carious lesions”*, and the subjects were divided into three groups: low risk (61-80%), medium risk (41-60%), and high risk (0-40%).
II: The patients were allocated into two groups, based on Cariogram scores at baseline. Each group consisted of low, moderate, and high risk subjects, which were revealed in the previous phase.
III: The intervention group received preventive programs (toothpaste containing 1,450 ppm fluoride, mouthwash, videos and plus photos encouraging oral health practices); and the control group was trained based on the routine oral health education by means of pamphlets and brochures.
IV: Cariogram parameters were calculated again for participants of both groups at the end of six months.

The standard Cariogram questionnaire was completed for all participants. Each of the nine caries-related factors was ranked from 0 to 2 or 0 to 3, based on the manual (Table 1). Then all data were entered into Cariogram program, in order to provide a graphic image to show the true chance of avoiding new caries cavities as percentages. The tenth factor (*‘‘clinical judgment”*) was given a score of 1 in all patients, which means that the caries risk was evaluated according to the other scores in the Cariogram. On the other hand, it shows the researcher’s agreement with the Cariogram program to evaluate caries’ risk in a normal condition.

**Table 1:** Caries-related factors used at baseline for the Cariogram.

Factors	Information and data collected	Cariogram scores
1 - Caries experience	Previous caries experience at baseline, including cavities, filling and missing teeth due to caries	0: Caries-free and no filling 1: Lower than the age group range 2: Within the age group range 3: Higher than the age group range
2 - Related diseases	General disease or conditions associated with dental caries, data from interviews and questionnaire	0: No disease, healthy 1: Disease/conditions, mild degree 2: Severe degree, long-lasting
3 - Diet, content	Estimation of the cariogenicity of the food, in particular fermentable carbohydrate content	0: Very low fermentable carbohydrate 1: Low fermentable carbohydrate 2: Moderate fermentable carbohydrate 3: High fermentable carbohydrate
4 - Diet, frequency	Estimation of number of meals and snacks per day (mean for a normal day)	0: Maximum 3 meals per day 1: Maximum 5 meals per day 2: Maximum 7 meals per day 3: More than 7 meals per day
5 - Plaque amount	Estimation of hygiene based on Silness-Loe plaque Index	0: PI < 0.4 (very good oral hygiene) 1: PI = 0.4 - 1.0 (good oral hygiene) 2: PI = 1.1 - 2.0 (poor oral hygiene) 3: PI > 2.0 (very poor oral hygiene)
6 - *Streptococcus mutans*	Estimation of levels of *Streptococcus mutans* in saliva using Strip mutans test (Orion Diagnostica Oy, Espoo, Finland)	0: 0 - 10^3^ CFU/ml saliva 1: 10 ^3^- 10^4^ CFU/ml saliva 2: 10 ^4^- 10^5^ CFU/ml saliva 3: > 10^5^ CFU/ml saliva
7 - Fluoride program	Estimation of the extent of fluoride available in the oral cavity, data from questionnaire	0: Receives ‘maximum’ fluoride program 1: Irregular but complete fluoride program 2: Fluoride toothpaste only 3: Avoiding fluorides, no fluoride
8 - Saliva secretion	Estimation of amount of saliva, using paraffin-stimulated saliva	0: more than 1.1 ml saliva/min 1: Low (0.9 - 1.1 ml stimulated saliva/min) 2: Low (0.5 - 0.9 ml saliva/min) 3: Very low (< 0.5 ml saliva/min)
9 - Saliva buffering capacity	Estimation of capacity of saliva to buffer acids	0: pH ≥ 6.0 1: pH 4.5 - 5.5 2: pH ≤ 4.0

### CARIOGRAM PROGRAM PARAMETERS

###  Caries experience 

The clinical examination was conducted in the orthodontic department of Shahid Beheshti dental school (Tehran/Iran), on a dental chair using mouth mirror, a standard light, and a dental probe. Caries was scored according to the World Health Organization (WHO) criteria, using DMFT index (number of decayed, missing, and filled teeth). 

Moreover, all oral examinations were performed by a single trained and calibrated researcher. Hence, only intraexaminer reliability was determined. Thus, the oral examination of 10 randomly selected subjects was repeated on different dates, to determine intraexaminer reliability. The Kappa coefficient value for intraexaminer reliability was 0.87, which is interpreted as very good.

**Figure 1:**
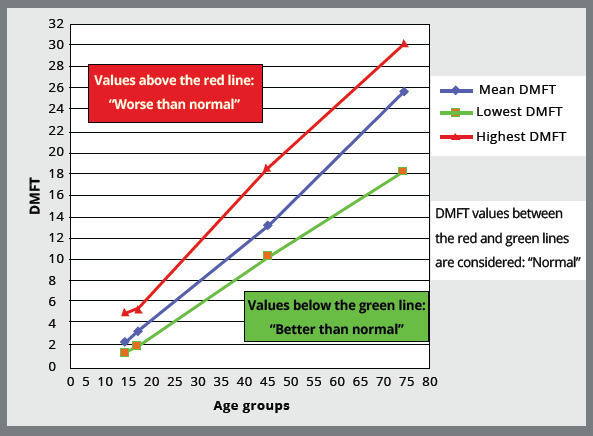
DMFT ( decayed, missing, and filled teeth ) values for different age categories, based on a national oral health survey performed in Iran in 2011. Source: Khoshnevisan et al.^14^, 2018.

In order to rank the current status of caries of the patients (first row of Table 1), the researchers need to know the caries prevalence in each country where the research was carried out. In collaboration with the oral health authorities, the previous history of caries was appointed, based on findings of a national oral health survey conducted in Iran in 2011^14^ (Fig 1). Thus, the condition of previous caries was rated from 0 to 3:

0. No decay or filling.
1. Better than normal: Green line or bellow, in Figure 1.
2. Normal for age group: Blue line, in Figure 1.
3. Worse than normal: Red line or above, in Figure 1.

####  Salivary flow rate 

Saliva was collected between 9:00 a.m. and noon, to minimize circadian rhythm effects, and at least an hour after drinking or eating food.

The steps to evaluate the amount of saliva flow were as follows:

» The patient should be seated in an upright and comfortable position.
» The patient should chew a paraffin pill for 30 seconds and then remove the stored saliva or swallow it.
» The patient should continue to chew for 5 minutes and accumulate saliva continuously in a sterile flask tube.
» After 5 minutes, the amount of saliva is measured, and the amount of stimulated saliva revealed (milliliter per minute) is given 0 to 3 points (according to row 8 in Table1).

####  Buffering capacity 

A 5 or 6-cm piece of litmus paper was placed in the test tube for 2 seconds. Once the color of the paper changed, the pH of the solution was deduced, by comparing the color of the paper with the color of the guide and, according to acid-base level, buffer capacity was determined as 0 to 2 points (according to row 9 in Table 1). 

####  Streptococcus mutans bacteria 

The samples of bacteria were collected using the saliva accumulated in the pre-sterilized single use containers, and were transferred to the laboratory at 4°C. The saliva was serially diluted and 0.1 ml was inoculated in a petri dish containing a dedicated culture medium (*Mitis salivarius*-bacitracin 10% sacarose agar). The dish was kept at 37°C for 48 hours in an incubator, for bacteria growth. Then, the colonies count was completed and, based on their number related to each milliliter of the saliva, a score of 0 to 3 was allocated to it.

All patients were examined by a single researcher. Data were collected according to the Cariogram program, i.e. medical and dental history, diet, dental plaque index, *Streptococcus mutans* and lactobacillus colony-forming units, fluoride intake, and the salivary samples to check the flow rate and its buffering capacity. After bonding the brackets, the clinical guidelines and oral routine recommendations were given to both groups of patients (intervention & control groups) by the therapist. An additional brochure about health education provided by the orthodontic department of Shahid Beheshti dental school was also given to them. The prevention programs were presented to the intervention group patients at the first session, after bracket placement. These programs were offered by the researcher as described below.

» Emphasis on the importance of regular dental care to check the white spot lesions, which are marks of initial tooth decay in patients.
» Nutritional counseling about how to change the diet, reduce the number of meals and snacks, consume fewer carbohydrates, and increase the amount of fiber foods.
» Delivery of a package containing the following four products:
  a) Oral-B Pro-Expert All-Around Protection Deep Clean 75 ml toothpaste, containing 1,450 ppm fluoride.
  b) Oral-B Pro-Expert Multi-Protection Mouthwash 250ml.
  c) Oral-B Super Floss.
  d) Oral-B interdental brush.
» Encourage regular brushing using the toothpaste provided with the package (two times in 24 hours, preferably in the morning before breakfast and at night before bedtime), and using the mouthwash included in the pack (two times daily, each time gargling for 30 seconds in the mouth).
» Presentation of films and photos related to proper brushing technique, interdental toothbrushes and Super Floss usage, by the researcher.

Determination of risk profiles (low / moderate / high) for each of the patients undergoing fixed orthodontic treatment was done through the Cariogram v. 3 (Malmo University, Sweden), which evaluates the given data based on its algorithms, and presents the results as a pie chart, indicating five different groups of factors related to dental caries, as follows:

1) Actual chance of avoiding new cavities: The green section shows an estimation of the “actual chance to avoid new cavities”. Patients are divided into three groups: high risk group (0-40%), moderate risk (40-60%) and low risk (60-100%) based on the percentage obtained from this section.
2) Diet: A dark blue section that shows the combined dietary content and its frequency.
3) Bacteria: The red part shows a combination of the amount of *Streptococcus mutans* and plaque.
4) Susceptibility: The light blue section shows a combination of three factors: the amount of fluoride intake; the amount of saliva secretion; and the saliva buffering capacity.
5) Circumstances: The yellow section is based on a combination of medical and dental history.

During the six-month study period, the researchers examined the patients who were in the intervention group, and asked them to demonstrate the correct use of toothbrushes and dental floss. The patients’ conditions were evaluated six months after starting orthodontic treatment. All data were collected again, and a Cariogram chart was drawn, to examine the outcome of the intervention. Mean values and standard deviation of the assessed indicators were reported in both intervention and control groups, at the beginning and at the end of the period. Due to the normal distribution of data, according to the Kolmogorov-Smirnov test, the independent sample *t*-test and Analysis of covariance (ANCOVA) were used to examine the effect of intervention on changes in the indices of the Cariogram and the differences between the two groups.

## RESULTS

The intervention group included 7 males and 11 females, and the control group consisted of 8 males and 10 females. The age range of participants was between 12 and 29 years, with the mean age of 19.6 ± 4.66 years and 19.28 ± 3.30 years in the intervention and control groups, respectively. 

**Figure 2:**
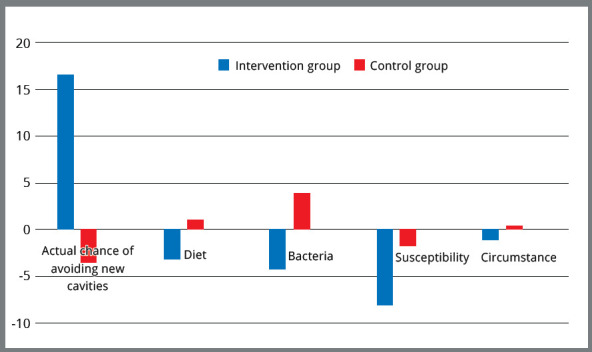
Cariogram parameters mean percentages between intervention and control groups, at the end of the study period (T1).

The various caries-related factors of Cariogram that were compared between the two groups at the beginning of treatment and after six months are shown in Table 2 and Figure 2. Results indicated an obvious increase in the percentages mean of *“Actual chance of avoiding new cavities”* section in the intervention group from 45.72 ± 21.64 to 62.50 ± 17.64, with a statistically significant difference (*p*< 0.001). At the baseline, the mean of other parameters such as “Diet”, “Bacteria” and “Susceptibility” was 17.50, 13.50, 16.89; and decreased to 14.28, 9.22, 8.78, respectively (*p*< 0.001). 

**Table 2:** Comparative of the Cariogram parameters mean percentages in both study groups, between baseline time (T0) and 6 months later (T1).

Cariogram parameters	Time	Control group		Intervention group		p-value
		Mean ± SD	Range	Mean ± SD	Range	
Actual chance of avoiding new cavities	T0	46.00 ? 21.11	15 - 87	45.72 ? 21.64	14 - 87	< 0.001
	T1	42.44 ? 19.45	14 - 80	62.50 ? 17.64	32 - 93	
Diet	T0	16.72 ? 7.19	5 - 30	17.50 ? 7.13	3 - 29	< 0.001
	T1	17.89 ? 5.98	6 - 30	14.28 ? 7.45	1 - 31	
Bacteria	T0	13.33 ? 8.36	2 - 30	13.50 ? 8.13	1 - 29	< 0.001
	T1	17.22 ? 8.46	5 - 37	9.22 ? 5.89	1 - 19	
Susceptibility	T0	17.33 ? 5.80	3 - 27	16.89 ? 6.79	4 - 29	< 0.001
	T1	15.56 ? 5.49	5 - 25	8.78 ? 4.68	1 - 17	
Circumstance	T0	6.61 ? 3.53	1 - 12	6.39 ? 3.88	1 - 13	< 0.001
	T1	6.89 ? 3.51	1 - 12	5.22 ? 3.62	1 - 12	

* Calculated by Analysis of Variance (ANOVA) test.

Independent sample *t-*test and covariance statistical method (ANCOVA) were used for analysis of the differences between baseline and after six months follow-up of both groups in the scores of the Cariogram parameters, and *p*-value < 0.05 was considered as significant. Also, there was significant difference between both study groups (*p*< 0.001), as illustrated in Table 3.

**Table 3:** The differences of the Cariogram parameters mean percentage between intervention and control groups, at the end of the study period (T1).

Cariogram parameters	Mean ± SD	Max	Min	*p* *
Actual chance of avoiding new cavities	20.33 ? 2.90	26.23	14.42	< 0.001
Diet	-8.16 ? 1.32	-5.48	-10.84	< 0.001
Bacteria	-4.38 ? 0.80	-2.75	-6.02	< 0.001
Susceptibility	-6.33 ? 1.24	-3.79	-8.87	< 0.001
Circumstance	-1.44 ? 0.38	-0.65	-2.23	< 0.001

* Calculated by independent *t-*test.

**Figure 3:**
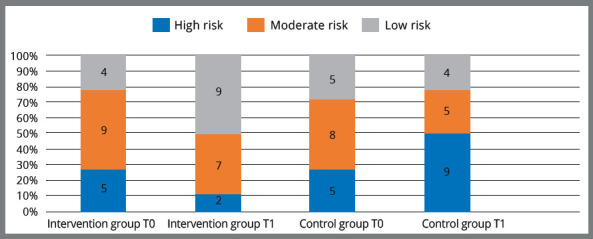
Intergroups differences in the distribution of the patients according to caries risk profiles, at the beginning of the study and after six months.

Furthermore, intervention group at the baseline was comprised of 22.2% (n = 4) low risk, 50% (n = 9) moderate risk, and 27.8% (n = 5) high risk. On the other hand, control group consisted of 27.8% (n = 5) low risk, 44.4% (n = 8) moderate risk, and 27.8% (n = 5) high risk patients. After six months, according to the Cariogram program, 11.2% (n = 2) displayed high caries risk, 38.8% (n = 7) displayed moderate caries risk, and 50% (n = 9) displayed low caries risk in intervention group. However, the distribution of the risk categories in control group was 50% (n = 9) high, 27.8% (n = 5) moderate, and 22.2% (n = 4) low caries risk (Fig 3).

## DISCUSSION

The present study focuses on the caries risk assessment in patients undergoing orthodontic treatment, and recommends preventive measures to reduce the occurrence of white spot lesions or new carious lesions. The results demonstrated a significant difference between the percentages of the Cariogram charts in the intervention and control groups, and also showed that the correct and regular use of standard toothpaste containing 1,450 ppm fluoride and standard mouthwash, using an interdental toothbrush and orthodontic floss along with proper and practical health education, can reduce the risk of dental caries - especially in highly susceptible (due to potential plaque accumulation) orthodontic patients. 

The present study showed that maintenance of improved oral health over longer time periods requires prolonged, repeated instructions, as explained by Zotti et al.^15^, who evaluated the influence of a mobile application-based approach for domestic oral hygiene maintenance in improving oral hygiene compliance and oral health, in a group of orthodontic patients. This study showed positive results in improving oral hygiene compliance of adolescent patients and in improving their oral health.^15^

By assessing the risk of caries using Cariogram, a significant reduction was observed in the intervention group patients, i.e., high caries risk subjects during the six months trial; also, the percentage of patients with moderate risk profile decreased. On the other hand, the percentage of patients with low risk profile increased in the intervention group. Karabekiroglu et al.^16^ reported that a period of twelve weeks is long enough to be able to detect preventive strategies; although other studies indicate that a period of at least six months is desired in order to identify the results of caries preventive methods. Hence, the present study was conducted for six months, being consistent with the above-mentioned research. During orthodontic treatment, due to the presence of orthodontic appliances in the mouth, it is possible to increase the chances of food being trapped and to increase the number of bacterial biofilms. There is no increased risk of caries under the orthodontic brackets, but there is increased risk around them, due to the plaque and food accumulation and poor oral hygiene. Good oral hygiene includes brushing teeth properly and regularly, as well as using mouthwash, dental floss and the interdental brush. Lack of appropriate oral hygiene in patients wearing fixed orthodontic appliances contributes to tooth decay, gingival recession, or discoloration of the teeth. The first sign of poor oral hygiene is often bleeding from gingival margins during the brushing. Individuals who neglect to take health/dental care during their treatment period face color changes around the brackets, with square or rectangular caries at the end of the treatment.^17^ Recent management of caries involves treating patients according to the risk (Low, Moderate, or High) and monitoring early lesions in tooth surfaces.

There have been many studies about increased caries risk in fixed appliances therapy, which has multiple factors in relation to orthodontic treatment, caries development, plaque accumulation, and effect of fluoride.^18^ ^,^ ^19^ In the present study, caries risk assessment using Cariogram indicated no single factor explaining the changes observed.

Also, the findings in the present study agree with the results of Mulla et al.^20^ study, which analyzes caries-related factors in patients undergoing fixed orthodontic treatment over a period of six months, using Cariogram, although in the present study the groups had undergone some interventions. The low caries group of that study displayed significantly lower decayed, filled surface index, lactobacillus and *Streptococcus mutans,* and plaque index after six months, and the percentage of the chance of avoiding new cavities was higher. Moreover, while another study concluded that Cariogram model can be used in orthodontic patients with or without the use of salivary tests,^21^ the current study analyzed all nine Cariogram parameters.

The efficacy of interventions for orthodontic white spot lesions (WSLs) has not yet been sufficiently evaluated in an evidence-based method; however, based on the review of interventions for post-orthodontic WSLs, monthly fluoride varnish use appears to be effective.^22^ Furthermore, Mannaa et al.^23^ concluded that the use of 5,000 ppm fluoride toothpaste for six weeks is able to reduce the caries risk, which can be clearly demonstrated using Cariogram program, due to increasing the actual chance of avoiding caries. They considered salivary lactobacillus counts as a measure of the cariogenic diet, which may indicate high carbohydrate consumption.^23^ In the present study, in addition to the level of lactobacillus, the frequency of meals per day (including snacks) was evaluated as a key factor in the estimation of caries risk, which may have a significant effect on the dietary component. In the present study, the scores of all Cariogram parameters decreased significantly in the intervention group after six months, compared to baseline levels.

Also, a decrease in the high caries risk profile due to increasing the actual chance of avoiding caries in the Cariogram pie chart, using 1,450 ppm fluoride toothpaste was confirmed in the Karabekiroglu et al.^16^ study, who used Cariogram to evaluate the effectiveness of 1,450 ppm fluoride toothpaste, fluoride varnish and chlorhexidine in adolescents for 12 weeks, and found no significant differences between the mentioned preventive methods. Likewise, Enerback et al.^24^ recommended the everyday use of high-fluoride toothpaste (5,000 ppm F) or mouth rinse (0.2% NaF), in combination with ordinary toothpaste, to reduce risk of caries during orthodontic treatment. However, saliva secretion and buffer capacity, which are two parameters in the Cariogram, were excluded from their study, due to time restraints and patient convenience - while all parameters of the Cariogram program were analyzed in the present study.

Almosa et al.^13^ used Cariogram to evaluate the factors related to caries between orthodontic patients treated in governmental and private centers immediately after orthodontic treatment. The results indicated that the percentage of an actual chance of avoiding new cavities in patients in public centers was lower than in private centers (28% and 61%, respectively). Also, DMFS, plaque index, number of *Streptococcus mutans* and lactobacillus, and salivary buffer capacity were significantly higher in the public group, compared with the private centers. The total number of caries lesions at debonding in the public group was more than two times higher than that in the private group.^13^

The current study was conducted only in a governmental, educational dental school, and supported the evidence related to caries risk assessment and individualized caries prevention strategies as an effective method of caries management. Further studies must compare large samples from different health centers (public and private clinics), with subjects in various situations (socio-economic status), to confirm the efficiency of preventive approaches for patients undergoing fixed orthodontic treatment. Besides, the present study had another limitation related to study design, that is, quasi-experimental design, which lacks true randomization. 

## CONCLUSION

Implementing different preventive strategies (using fluoridated toothpaste and mouthwash, educational videos and images) is useful to decrease caries risk in patients undergoing fixed orthodontic treatment. This issue is also shown by increasing *“Actual chance of avoiding new cavities”* section in Cariogram program.
